# Thermal Cavitation Effect on the Hydrodynamic Performance of Spiral Groove Liquid Face Seals

**DOI:** 10.3390/ma17112505

**Published:** 2024-05-23

**Authors:** Yuansen Song, Shaoxian Bai

**Affiliations:** Institute of Process Equipment and Control Engineering, College of Mechanical Engineering, Zhejiang University of Technology, Hangzhou 310032, China; songyuansen0704@163.com

**Keywords:** thermal cavitation effect, liquid lubrication, hydrodynamic performance, cavitation, liquid face seals

## Abstract

Cavitation in micro-scale lubricating film could be determined by the fluid’s thermal properties, which impacts the hydrodynamic lubrication capacity dramatically. This study aimed to novelly investigate the impact of the thermal cavitation effect on the hydrodynamic performance of liquid face seals, employing the compressible cavitation model, viscosity–temperature effect, and energy equation. The finite difference method was adopted to analyze the thermal cavitation by calculating the pressure and temperature profiles of the lubricating film. The working conditions and geometric configuration of liquid face seals under different thermal cases were further studied to explore their effects on sealing performance. The results showed that thermal cavitation could reduce the temperature difference of liquid film at high speeds, and cavitation would be weakened under temperature gradients, which further dropped off the hydrodynamic performance. Contrary to the leakage rate, the opening forces tended to be lower with the increasing seal pressure and film thickness under high-temperature gradients. Furthermore, apart from the spiral angle of grooves, the hydrodynamic performance exhibited significant variation with increasing groove depth, number, and radius at high-temperature gradients, which meant that the thermal cavitation effect should be considered in the design of geometric grooves to obtain better hydrodynamic performance.

## 1. Introduction

The liquid face seal [1,2,3] (LFS), serving as a critical element in rotary power machinery, can ensure the long life and reliable operation of the equipment as a result of the hydrodynamic performance of lubricating film improved by the surface textures (such as spiral grooves [4], dimples [5], etc.). Meanwhile, cavitation is a complex mechanism in the lubrication analysis due to various inducing factors and the weakened hydrodynamic performance produced, especially at high speeds [6,7]; and its complexity further increases when the thermal properties of the cavitation medium are involved [8,9]. In the current engineering practice, the equipment gradually develops for high working parameters (high speed, high temperature, etc.), and the thermal cavitation effect cannot be ignored to better predict the serviceability and optimize the design.

In fact, the behavior of the seal can be significantly influenced by thermal effects at high rotation speeds. For example, the utilization of thermocouples integrated within the stationary ring, as demonstrated by Denny [10] in 1961, revealed a significant increase in facial temperature by several tens of degrees. In addition, Orcutt [11] employed a pyrometer to conduct temperature measurements of the rotor surface. The observed temperature gradient along the radial direction could exceed 10 K. The above findings were subsequently validated by Tournerie et al. [12] through the utilization of thermography as a technique. As observed by Doust and Parmar [13], a small inclination of the sealing ring happened due to the variations in surface temperature, resulting in their acquisition of a tapered shape. This conical deformation significantly contributed to the transformation of the sealing surface‘s lubricating state. Therefore, it is essential to consider thermal effects in thermo-hydrodynamic performance analysis.

However, the physical fields of the sealing ring and lubricating film (such as temperature, pressure, etc.) are likely to undergo significant changes when combined with the thermal effect and cavitation effects. Generally, the appearance of cavitation within a lubricating film happens as gas or vapor continuously separates the liquid phase [14,15,16]. This is an unavoidable phenomenon in liquid lubrication after the pressure decreases to levels less than the cavitation pressure for the liquid film [17,18,19,20,21,22]. In this process, the thermal effect is often overlooked, and the temperature is consistently assumed to be uniform throughout the entire flow field. Actually, the cavitation also affects the temperature distribution of the sealing surface. In 2015, a study was carried out by Migout [23] on the vaporization of fluid film at the sealing surface. It was suggested that the existence of a phase transition could result in a notable decline in the surface temperature. For instance, when the sealed medium’s temperature was 165 °C, the cooling effect from phase transition was effective enough to bring down the contact temperature below that level. However, when subjected to viscous shear and temperature gradient conditions, a discernible temperature variance may manifest within the lubricating film, bringing about alterations in the physical properties of the lubricant that subsequently impact cavitation [24,25]. Several studies [26,27,28,29] have been conducted on the thermal cavitation effect on turbopump inducers, demonstrating that the role of thermal is essential in inhibiting the expansion of cavitation bubbles, as it acts as a major mechanism for dissipating energy. In essence, it has the capability to inhibit cavitation occurrence under thermal conditions. However, limited research has been conducted on the impact of thermal effects on cavitation within micron-scale clearances.

In the theoretical analysis of cavitation, Marian et al. [30] pointed out that the model ensuring the mass conservation of the hydrodynamic flows is the priority to obtain reliable predictions. The mass-conserving Jakobsson-Floberg-Olsson (JFO) cavitation model typically assumes constant pressure throughout the entire cavitation region [31,32,33,34]. Nevertheless, Etsion and Ludwig [35] demonstrated a noteworthy 50 kPa-pressure fluctuation in the cavitated zone while conducting visual experiments on journal bearings. Our recent research [36] has introduced a new cavitation model that considers the pressure variation within the cavitation zone, which aligns well with Etsion’s experimental findings [35]. As described above, the temperature field of the sealing surface interacts with the cavitation. Hence, it is crucial to further analyze the distribution of pressure in regions experiencing cavitation within the context of liquid thermo-hydrodynamic lubrication.

The presence of surface grooves is crucial for enhancing the thermo-hydrodynamic performance, based on the thermal cavitation effect. In 2018, a study on a mechanical face seal by Adjemout M. et al. [37] investigated surface texturing’s effect on tribological properties and temperature, both theoretically and experimentally. The results showed a 50% decrease in friction across all speeds and a decrease in the seal face’s temperature rise, with the maximum velocity experiencing a 40% decrease. Xue et al. [38] optimized the shape structure and lines of the groove and carried out visualization experiments at 500 rpm and 1000 rpm. The findings indicated that the altered geometric configuration of the groove would effectively inhibit cavitation. However, further research is needed to analyze the synthetic impact of groove structure and thermal cavitation on the performance of LFS at temperature gradients and high speeds.

This paper novelly focuses on the influence of the thermal cavitation effect on the hydrodynamic efficiency of spiral groove liquid face seals when subjected to high speeds and temperature gradients. By employing the compressible cavitation model, viscosity–temperature effect, and energy equation, we obtained temperature and pressure profiles to determine the thermal cavitation. The working parameters (including seal pressure, rotation speed, film thickness, lubricant temperature, etc.) and geometric configuration (such as groove depth, groove number, spiral groove, and grooved radius) under different thermal cases are further studied to explore its effects on the sealing performance.

## 2. Modeling and Numerical Methods

### 2.1. Geometry Characterization

**Figure 1:**
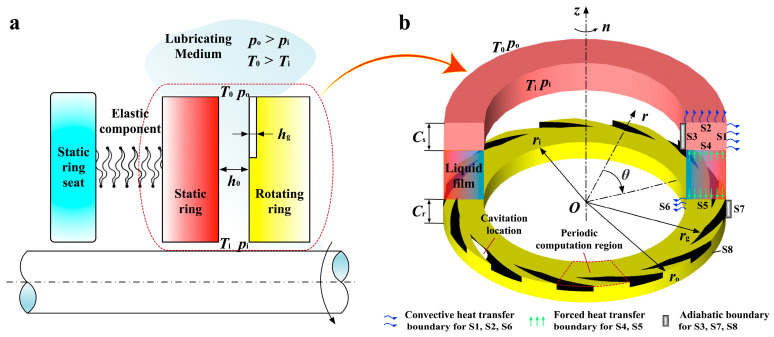
Schematic diagram of liquid face seals. (**a**) Basic sealing components; (**b**) Structure of sealing rings and boundary conditions.

The thermo-hydrodynamic lubrication with cavitation effect of the liquid face seal is taken into account, as presented in Figure 1. The inner radius *r*_i_ of the seal has a temperature of *T*_i_ and a pressure of *p*_i_. Because of the periodical spiral groove with a depth of *h*_g_, the lubricants with the temperature *T*_0_ and pressure *p*_o_ at the outer radius *r*_o_ are transported to the clearance to develop a liquid film with the thickness *h*_0_, where the cavitation inevitably happens in the texturing. The film thickness of LFS could be expressed as follows:

(1) $$h(r,\theta )=\left\{\begin{array}{ll} h_{0} & \mathrm{non}\text{-}\mathrm{grooved}\;\mathrm{region}\\ h_{0}+h_{g}(r,\theta ) & \mathrm{grooved}\;\mathrm{region} \end{array}\right.$$

Figure 1b depicts the thermal boundary conditions of the lubricating medium and sealing ring. The static ring and rotating ring have thicknesses of *C*_s_ and *C*_r_, respectively. S1, S2, and S6 are convective heat transfer boundaries. S4 and S5 have forced heat transfer boundaries. S3, S7, and S8 have adiabatic boundaries.

Geometric configuration parameters and working parameters for LFS with spiral grooves are listed in the following Table 1 and Table 2, respectively.

### 2.2. Analytical Methods

#### 2.2.1. Mathematical Formulations

Considering the liquid film cavitation, the Reynolds equation applicable for analyzing the lubrication of LFS under steady-state conditions [39] is shown as follows:

(2) $$\frac{\partial }{\partial r}\left(\frac{\rho rh^{3}}{\eta }\frac{\partial p}{\partial r}\right)+\frac{\partial }{\partial \theta }\left(\frac{\rho h^{3}}{\eta }\frac{\partial p}{r\partial \theta }\right)=6\omega r\frac{\partial (\rho h)}{\partial \theta }$$

where the symbols *η* and *ρ* represent the local viscosity and density of the lubricating medium, respectively; *ω* and *p* refer to the angular velocity of the sealing ring and local pressure, respectively.

In cavitation analysis, fluid compressibility is considered. Assuming that the medium is present as a fully gas phase in the cavitated area, cavitation pressure *p*_c_ is the critical pressure of phase transformation, and the density at this time is equal to the density in the liquid state. Therefore, the subsequent equation can be utilized to elucidate the phenomenon of cavitation [36].

(3) $$\rho =\left\{\begin{array}{ll} \rho_{0} & \mathrm{full}\;\mathrm{film}\;\mathrm{region}\;(p>p_{c})\;\\ p_{c}\rho_{0}/p & \mathrm{cavitation}\;\mathrm{region}\;(p\leq p_{c}) \end{array}\right.$$

where *ρ*_0_ indicates the density in the liquid state.

Pressure distribution inside the cavitation zone will be defined through the above cavitation model. The minimum pressure of the film further demonstrates the ability of phase change. So, the ratio between *p*_min_ and *p*_c_ of the lubricating film could be utilized for additional characterization of cavitation intensity [40], as expressed below.

(4) $$S=\frac{p_{c}-\min\left\{p\right\}}{p_{c}}$$

The energy equation [39] for the thermo-hydrodynamic lubrication analysis could be written as follows:

(5) $$q_{r}\frac{\partial T}{\partial r}+q_{\theta }\frac{\partial T}{r\partial \theta }=\frac{\eta \omega^{2}r^{2}}{h\rho c_{v}}-\frac{h^{3}}{\eta \rho c_{v}}\left[{\left(\frac{\partial p}{\partial r}\right)}^{2}+{\left(\frac{\partial p}{r\partial \theta }\right)}^{2}\right]+\frac{k_{\mathrm{ls}1}}{\rho c_{v}}(T_{s1}-T)+\frac{k_{\mathrm{ls}2}}{\rho c_{v}}(T_{s2}-T)$$

where *T*_s_ represents the seal ring’s temperature; *T* denotes the lubricating film’s local temperature; *k*_ls_ represents the convective heat transfer coefficients; *c*_v_ refers to the lubricant’s specific heat; subscripts 1 and 2 denote the rotating and stationary ring, correspondingly; and *q*_r_ and *q*_θ_ are circumferential and radial flow rates, respectively [39].

(6) $$q_{r}=-\frac{rh^{3}}{12\eta }\frac{\partial p}{\partial r}$$

(7) $$q_{\theta }=-\frac{h^{3}}{12\eta }\frac{\partial p}{r\partial \theta }+\frac{\omega rh}{2}$$

The impact of thermal effects necessitates consideration of the lubricant’s viscosity–temperature properties. Here, Barus’s viscosity equation [39] is adopted as follows.

(8) $$\eta =\eta_{0}\exp[-\tau (T-T_{0})]$$

where *η*_0_ refers to the lubricant viscosity in the initial state; *τ* denotes viscosity–temperature coefficient.

The convective heat transfer coefficients *k*_ls_ [39] could be written as follows.

(9) $$k_{\mathrm{ls}}=\frac{3{\left(c_{p}\eta \right)}^{1/3}k_{c\_\mathrm{liq}}^{2/3}}{h_{0}}$$

where *k*_c_liq_ and *c*_p_ refer to the fluid’s heat conduction and specific heat.

Further, the heat conduction equations for the sealing ring are shown as follows [39]:

(10) $$\frac{\partial }{r\partial r}\left(r\frac{\partial T_{s}}{\partial r}\right)+\frac{\partial^{2}T_{s}}{r^{2}\partial \theta^{2}}+\frac{\partial^{2}T_{s}}{\partial z^{2}}=\frac{\rho_{s2}c_{s2}\omega }{k_{c2}}\frac{\partial T_{s}}{\partial t}$$

(11) $$\frac{\partial }{r\partial r}\left(r\frac{\partial T_{s}}{\partial r}\right)+\frac{\partial^{2}T_{s}}{r^{2}\partial \theta^{2}}+\frac{\partial^{2}T_{s}}{\partial z^{2}}=0$$

where *ρ*_s_, *k*_c_, and *c*_s_ stand for the sealing ring’s density, heat conduction, and specific heat, correspondingly.

The boundary conditions primarily consist of pressure, liquid film temperature, and solid heat transfer. The pressure conditions could be expressed as:

(12) p(r,θ=π/Z)=p(r,θ=−π/Z)

(13) $$p(r=r_{i},\theta )=p_{i},\;p(r=r_{o},\theta )=p_{o}$$

The liquid film temperature conditions [39] could be expressed as:

(14) T(r,θ=π/Z)=T(r,θ=−π/Z)

(15) $$\left\{\begin{array}{ll} T(r=r_{o},\theta )=T_{o} & \mathrm{if}\;q(r=r_{o},\theta )<0\\ T(r=r_{i},\theta )=T_{i} & \mathrm{if}\;q(r=r_{o},\theta )>0 \end{array}\right.$$

The forced heat transfer equation [39] could be expressed as:

(16) $$-k_{c1}{\left(\frac{\partial T_{s}}{\partial n}\right)}_{s}=k_{\mathrm{ls}1}(T_{s1}-T)$$

(17) $$-k_{c2}{\left(\frac{\partial T_{s}}{\partial n}\right)}_{s}=k_{\mathrm{ls}2}(T_{s2}-T)$$

The characteristics of the lubricant and sealing ring used [36,40,41] in the analysis can be found in Table 3 and Table 4.

Based on considering thermal and cavitation, its influence is mainly considered from two aspects of seal performance: leakage rate *q* and opening performance *F* [39].

(18) $$F=\int_{0}^{2\pi }\int_{r_{i}}^{r_{o}}prdrd\theta$$

(19) $$q=-\frac{1}{12\eta }\int_{0}^{2\pi }h^{3}r\frac{\partial p}{\partial r}d\theta$$

In the following computation, the nondimensionalized pressure will be expressed as:

(20) $$P=p/p_{a}$$

#### 2.2.2. Numerical Techniques and Procedure

**Figure 2:**
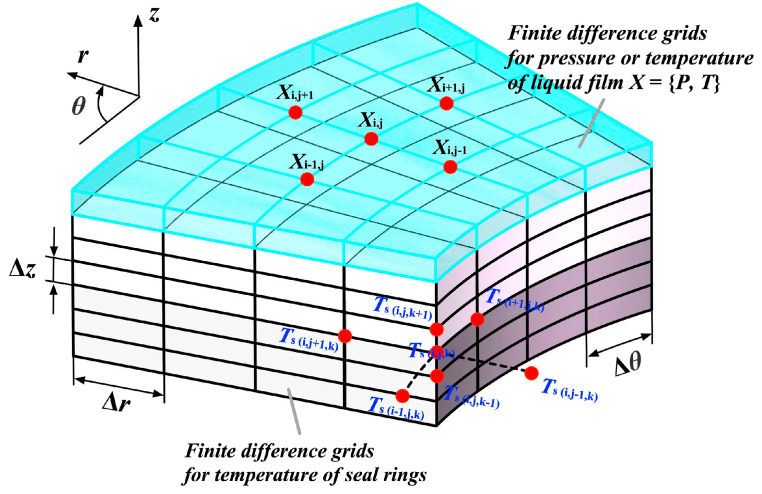
Schematic diagram of finite difference grids for seal rings and liquid film in the numerical simulation of pressure and temperature (Blue sky lines represent the finite difference grids of liquid film, black lines represent the finite difference grids of seal rings, red dots represent the grid nodes).

Numerical computation makes use of finite difference methods to solve the governing equations for determining pressure and temperature profiles through the relaxation iteration method [39,42]. To simplify the computation, one period of the model will be chosen, and the corresponding meshed domains are depicted in Figure 2. Here, the grid density is 60 × 61 for liquid film, and that of rings is 60 × 61 × 33.

**Figure 3:**
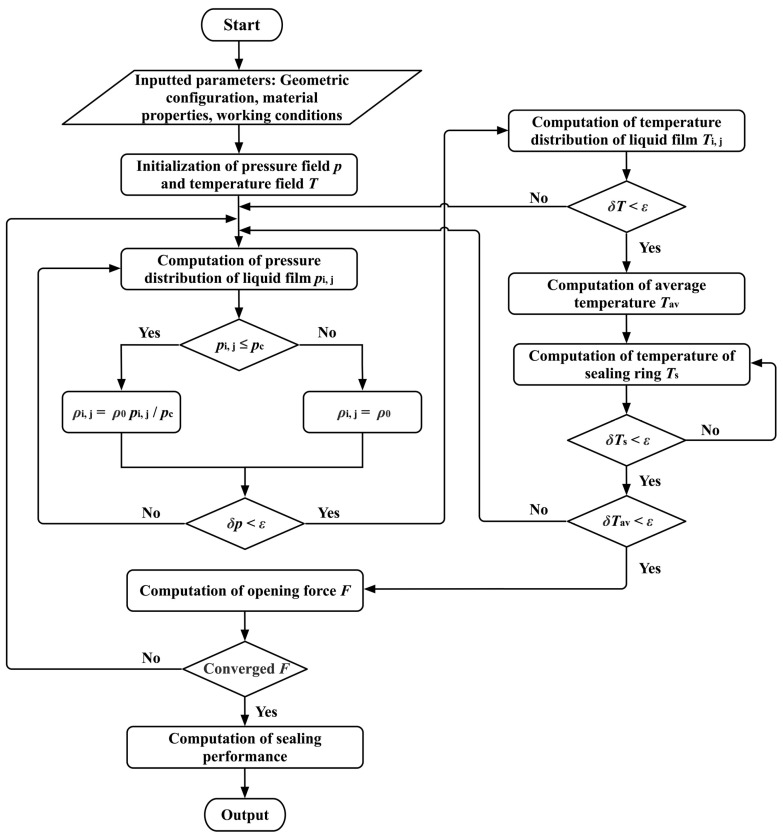
Flow chart of numerical calculation of mathematical model.

Figure 3 depicts the numerical computation process for the mathematical model. First, the fundamental parameters (including the structural parameters, working conditions, and material properties of LFS) can be initially entered. Referring to the governing equation for lubrication, the initial calculation involves determining the pressure field *P* and temperature field *T*. The iteration continues until the convergence criterion (*δp* < *ε*) is met.

When the coupling effect of thermal and cavitation is taken into account, the temperature distributions *T*_i,j_ are obtained after the pressure fields satisfy the convergence criterion. The seal rings’ temperature field *T*_s_ and the lubricating film’s mean temperature *T*_av_ can be calculated after satisfying the *δT* < *ε*. When the above two temperatures converge (i.e., *δT*_s_ < *ε*, *δT*_av_ < *ε*), the calculation of thermo-hydrodynamic lubrication considering the thermal cavitation effect is completed. Further, the whole iterative calculation process ends when the opening force *F* converges. On this basis, all parameters are obtained, and the results are output.

During the iterative process of the numerical solution, a convergence criterion is established with an error limit *ε* set at 10^−5^. The convergence criterion can be expressed in the following equation:

(21) $$\delta Y=\left|\frac{Y^{m}-Y^{m/2}}{Y^{m}}\right|$$

where *Y* = {*p*, *T*, *T*_s_, *T*_av_, *F*}, *m* is the number of iterations.

For the validation of numerical modeling, in our previous works [36,40], the experimental cavitation phenomena of submerged journal bearings by Etsion et al. [35] and the thrust bearings with texture surfaces by Zhang and Meng [5] were compared and analyzed, which verified the effectiveness of this cavitation model in the calculation of pressure inside the cavitation zone and in the analysis of lubrication of texture surfaces. In addition, in another work [43], the experimental work of the porous face seal surface temperature rise by Dingui and Brunetiere et al. [44] was further compared and analyzed, which verified the effectiveness of the model in the analysis of thermo-hydrodynamic lubrication.

## 3. Thermal Cavitation Effect

Due to the friction heat generated by the surface contact, a temperature gradient of order 1–10 K·mm^−1^ could be produced, which may affect the properties of the lubricating medium and further affect the lubrication performance [45,46]. Therefore, the following sections focus on the analysis of the pressure distributions, cavitation intensity, and temperature distributions under different thermal environments to analyze the thermal cavitation effect.

### 3.1. Pressure Distributions

**Figure 4:**
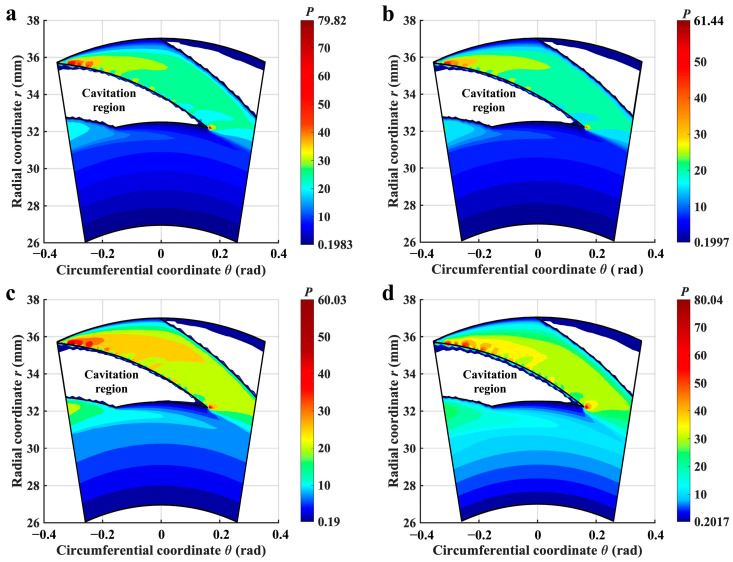

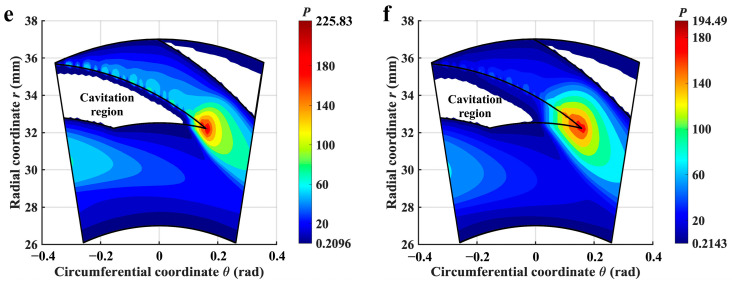
Contour of liquid film pressure distributions in different thermal cases (*Z* = 12, *h*_g_ = 6 μm, *β* = 18°, *r*_g_ = 32.5 mm, *h*_0_ = 5 μm, *p*_o_ = 0.3 MPa, *n* = 15,000 rpm, *T*_i_ = 300 K). (**a**) represents the case without consideration of thermal; (**b**) represents the isothermal case with ∇*T* = 0 K·mm^−1^; (**c**–**f**) show the thermal cases with ∇*T* = 1.3, 3.3, 5.3, and 7.3 K·mm^−1^, respectively.

In Figure 4, a comparison between the lubricating film’s pressure distributions under non-thermal and thermal cases has been conducted to investigate the impact of thermal cavitation. Compared to the isothermal condition (the temperature at two sides of the seal face is 300 K, as depicted in Figure 4b), the pressure field in the non-thermal case (as shown in Figure 4a) tends to exhibit higher film pressure, indicating that thermal effects weaken the hydrodynamic effect. Furthermore, Figure 4c,d displays the contour of the pressure fields for ∇*T* values of 1.3, 3.3, 5.3, and 7.3 K·mm^−1^, respectively. It can be noted that, as the temperature gradient increases, the size of the cavitation zone within the grooved region decreases at a constant temperature of 300 K at the inner radius. Meanwhile, the dimensionless peak pressure of the lubricating film, *P*_max_, increases in accordance with this trend. When ∇*T* increases to 5.3 K·mm^−1^ from 1.3 K·mm^−1^, *P*_max_ goes from 60.3 to 225.83 (nearly a fourfold increase). Then, when it rises to 7.3 K·mm^−1^, the maximum pressure begins to decrease.

The primary factor contributing to the shrinking cavitation and change of pressure distribution is the decrease in viscosity of the lubricating medium caused by increasing temperature gradients, leading to a reduction in viscous shear under high-velocity conditions. According to reference [47], the development of the cavitation region is primarily influenced by the shear effect, and a decrease in the strength of the shear effect will result in a reduction of the cavitation region, as shown in Figure 4c,f. The reduction of the cavitation area is more favorable for the pumping action of the spiral groove. More lubricating medium is pumped to the sealing face, extruded, and accumulated near the groove root, so there is a high-pressure zone near it, which is also the reason for the gradual increase in *P*_max_. The reason for the decrease in the maximum pressure is mainly caused by the thermal-viscous effect of the higher medium temperature that cannot promote the extrusion of the liquid and generate enough hydrodynamic effect. So, it can be inferred that the thermal cavitation effect has the potential to attenuate the formation of cavitation and promote the pumping effect of surface grooves but to some extent drop off the hydrodynamic effect of the lubricating medium.

### 3.2. Cavitation Intensity

**Figure 5:**
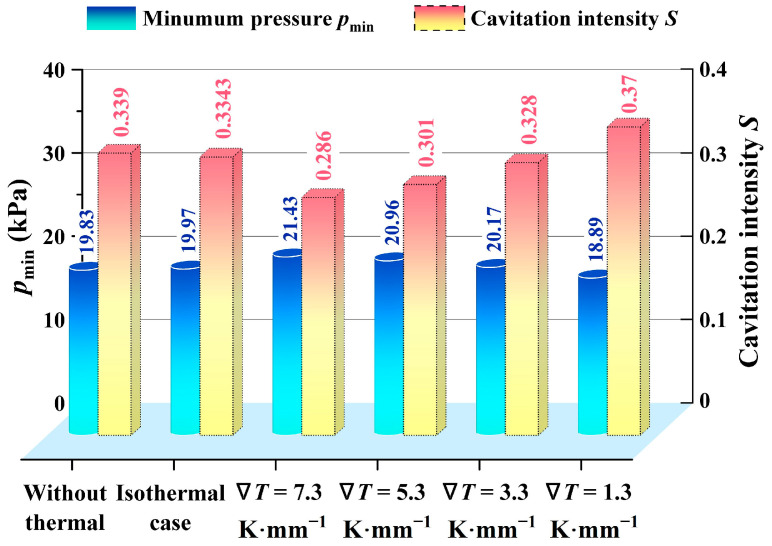
The minimum pressure *p*_min_ and cavitation intensity *S* in different thermal cases (*Z* = 12, *h*_g_ = 6 μm, *β* = 18°, *r*_g_ = 32.5 mm, *h*_0_ = 5 μm, *p*_o_ = 0.3 Mpa, *n* = 15,000 rpm, *T*_i_ = 300 K).

In Figure 5, the variation of the cavitation intensity *S* influenced by the thermal effect is illustrated in the isothermal case, thermal case, and the case without thermal. The intensity of cavitation is closely associated with both *p*_c_ and *p*_min_ of the lubricating film. The greater the minimum pressure, the higher the intensity of cavitation, indicating that the lubricating film is more susceptible to cavitation. It is evident that *p*_min_ in the isothermal case and without a thermal case is relatively bigger than in the thermal cases with a temperature gradient. Hence, the cavitation intensity is smaller in the high-temperature gradient. For example, when the ∇*T* increases from 1.3 to 7.3 K·mm^−1^, the cavitation intensity decreases by about 23.5%.

The main reason for the above phenomenon Is that the increasing temperature tends to contribute to the decrease of lubricating film viscosity, which will theoretically lead to the weakening of the cavitation effect. As the temperature gradient increases, the shrinking cavitation zone will enhance the groove’s ability to pump more medium and create a high-pressure zone with higher pressure. This, in turn, limits the liquid film phase transition ability to some extent. Consequently, the minimum pressure in the cavitation zone continues to rise, leading to a gradual decrease in cavitation intensity.

### 3.3. Temperature Fields

**Figure 6:**
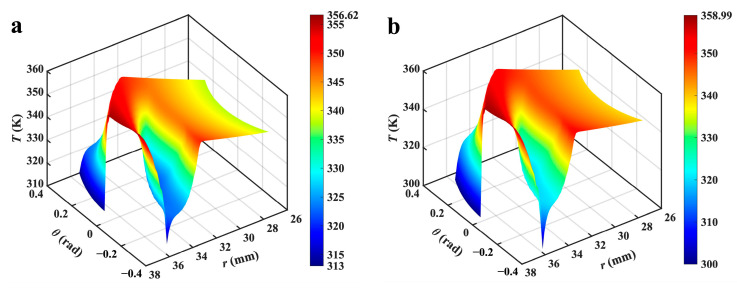

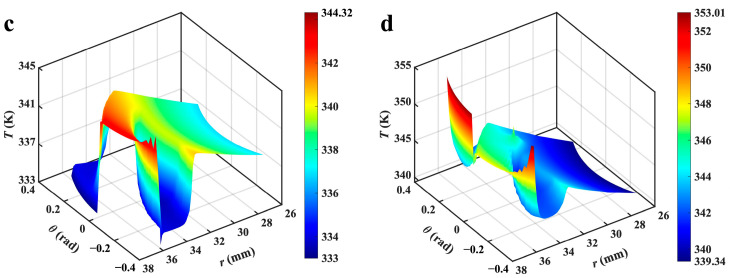
Contour of temperature fields of liquid film in different thermal cases (*Z* = 12, *h*_g_ = 6 μm, *β* = 18°, *r*_g_ = 32.5 mm, *h*_0_ = 5 μm, *p*_o_ = 0.3 MPa, *n* = 15,000 rpm, *T*_i_ = 300 K). (**a**–**d**) show the thermal cases with ∇*T* = 0, 1.3, 3.3, and 5.3 K·mm^−1^, respectively.

Figure 6 presents the contour of the temperature fields of the liquid film in different thermal cases. Except for the area affected by cavitation, the temperature profiles exhibit a decreasing trend along the direction of positive pressure differential of mediums. The cavitation region’s temperature would be notably lower compared to the liquid film at high velocities, this is mainly because of the fluid expansion and heat absorption in this region under the action of thermal cavitation. Furthermore, the peak temperature on the surface typically occurs near the outer diameter and rises in conjunction with the temperature of the lubricating medium. However, when ∇*T* is 5.3 K·mm^−1^, the decrease in the cavitation area leads to a quick temperature drop close to the outer edge of the sealing surface, especially in the spiral grooves’ outer region. Additionally, there is a relatively high temperature near the groove root. The temperature rise in the vicinity of the high-pressure area within the liquid film is primarily attributed to fluid shear and high-speed, high-temperature extrusion.

**Figure 7:**
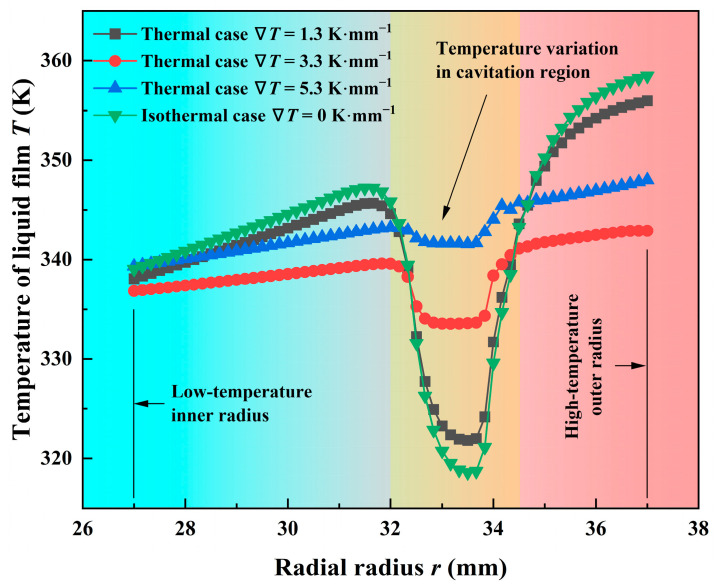
Temperature profiles of liquid film at the radial section along the center line of the computational domain in different thermal cases (*Z* = 12, *h*_g_ = 6 μm, *β* = 18°, *r*_g_ = 32.5 mm, *h*_0_ = 5 μm, *p*_o_ = 0.3 MPa, *n* = 15,000 rpm, *T*_i_ = 300 K).

To further explore the thermal cavitation effect under different thermal conditions, the temperature profiles along the center line of the computational domain are analyzed in Figure 7. The liquid film temperature under isothermal cases is larger than that under the other three thermal cases in the non-cavitation region, but the temperature in the cavitation region is lowest under isothermal cases (about 319 K). Moreover, when the temperature gradient along the radius direction from outlet to inlet of the lubricant rises from 0 to 3.3 K·mm^−1^, the temperature difference of liquid film ∆*T* between the inner and outer radius decreases. ∆*T* is 19.43 K in the isothermal case, while it decreases to 6.05 K in the thermal cases ∇*T* = 3.3 K·mm^−1^, there is a about 68.9% decline. While ∇*T* continues to increase, ∆*T* happens to grow up slightly. Therefore, it is known that thermal cavitation can reduce the temperature difference of the sealing face to a large extent, which has a certain effect on the wedge thermal deformation.

The above phenomenon is mostly because the inside of the grooves is almost completely cavitation under high-speed conditions, as shown in Figure 4a. At this time, bubbles in the liquid will expand and absorb heat, resulting in a temperature decrease. Meanwhile, the limitation of the pumping action of the grooves affects the heat exchange of the liquid film, so the temperature in the cavitation zone drops greatly under isothermal conditions. In addition, the rapid changes in pressure and volume as bubbles collapse can lead to adiabatic cooling, where no heat is exchanged with the surroundings, but the temperature drops due to the expansion of gases within the bubbles.

**Figure 8:**
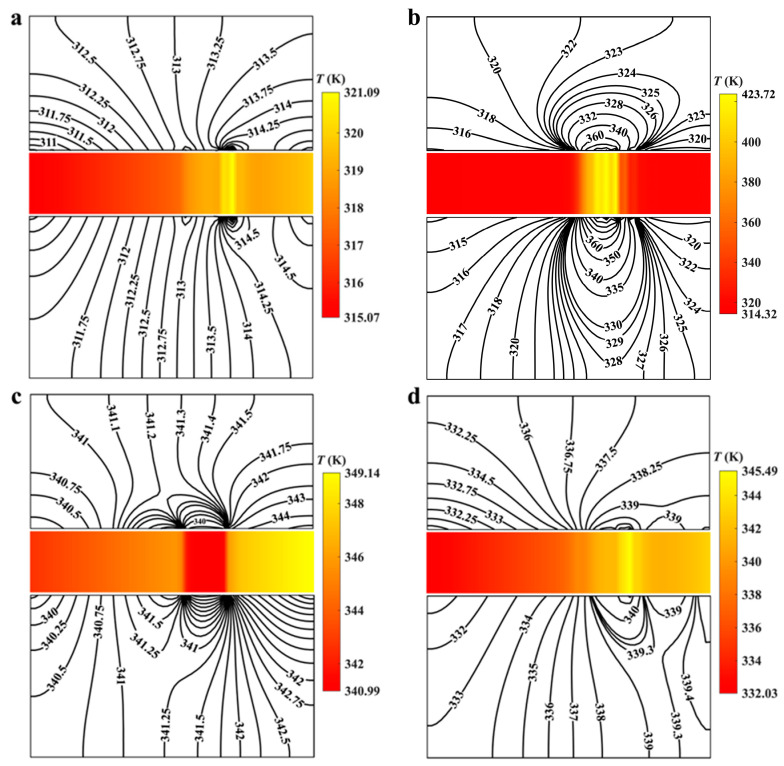
Temperature profiles of liquid face seals in the radial section at different speeds and temperature gradients (*Z* = 12, *h*_g_ = 6 μm, *β* = 18°, *r*_g_ = 32.5 mm, *h*_0_ = 5 μm, *p*_o_ = 0.3 MPa, *T*_i_ = 300 K). (**a**) ∇*T* = 4.3 K·mm^−1^, 7500 rpm; (**b**) ∇*T* = 7.3 K·mm^−1^, 7500 rpm; (**c**) ∇*T* = 4.3 K·mm^−1^, 15,000 rpm; (**d**) ∇*T* = 7.3 K·mm^−1^, 15,000 rpm.

The fluctuation in temperature of the lubricating film will directly affect the sealing ring’s temperature, which further affects the mechanical properties. The temperature profiles of LFS (including liquid film and sealing rings) in the radial section at various speeds and temperature gradients are depicted in Figure 8, aiming to further investigate the impact of thermal cavitation.

In general, the liquid film is more prone to cavitation at high speeds. However, according to the above, the increase in temperature leads to a decreasing cavity, and the size of that affects the temperature change of the lubricating film. At the speed of 7500 rpm (as shown in Figure 8a,b), when ∇*T* increases from 4.3 to 7.3 K·mm^−1^, the temperature difference of the liquid film decreases from 4.8 K to 4.26 K, while that of the rotating ring decreases from 4.48 K to 3.9 K, and that of the static ring decreased from 4.46 K to 3.54 K. However, when the rotating speed is doubled to 15,000 rpm (as shown in Figure 8c,d), the temperature difference of liquid film increases by about 70% from 6.5 K; and the temperature difference of rotating ring increases by about 39.3% from 6.1 K; the temperature difference in the static ring increases by about 61% from 5.9 K. Therefore, at relatively low speeds, the thermal cavitation effect can reduce the temperature difference on the seal surface However, although the lubricant viscosity decreases and then the cavitation strength decreases, the high viscous shear effect and the high-temperature gradient will still cause the increase in the surface temperature difference of the sealing ring.

## 4. Sealing Performance Analysis

The performance of LFS could be distinguished by analyzing the primary factors of opening force and leakage rate. Next, the impact of the variations in both geometric structures of seal rings and the working conditions of the seals on them are discussed under the influence of thermal cavitation of the lubricating medium.

### 4.1. Influence of Working Conditions

#### 4.1.1. Lubricant Temperature

**Figure 9:**
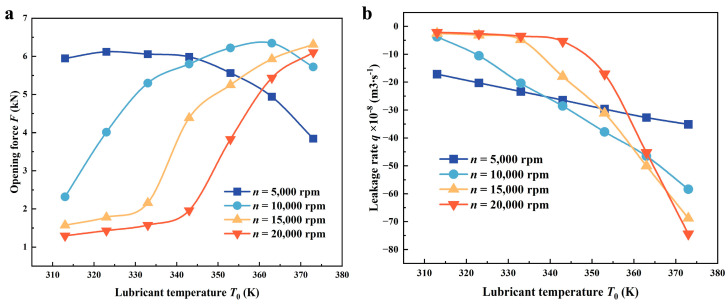
Variations of (**a**) opening force and (**b**) leakage rate as a function of lubricant temperature *T*_0_ under various thermal conditions (*Z* = 12, *h*_g_ = 6 μm, *β* = 18°, *r*_g_ = 32.5 mm, *h*_0_ = 5 μm, *p*_o_ = 0.3 MPa, *T*_i_ = 300 K).

The opening force of the seals is plotted against the temperature of lubricant *T*_0_ in Figure 9a. At multiple rotation speeds, *F* presents different variation tendencies with the increase of *T*_0_. First, *F* increases firstly and declines gradually at the speeds of 5000 rpm and 10,000 rpm. But the critical temperature at which *F* begins to decrease tends to be higher at high speed. At 5000 rpm, the temperature is about 320 K, while at 10,000 rpm, the temperature increases to 360 K, a temperature increase of about 12.5%. Furthermore, *F* shows a continuously increasing tendency with a speed higher than 15,000 rpm when *T*_0_ increases from 313 K to 373 K. The leakage rate variation shown in Figure 9b indicates that *q* increases at various speeds with the increase of *T*_0_, but at higher speeds, the temperature of the lubricating medium causing the leakage rate to increase is relatively higher. At higher temperatures and speeds, the leakage rate is often larger. For example, compared to a speed of 15,000 rpm, the leakage rate sharply drops at 20,000 rpm for a temperature of 343 K with a temperature increase of about 20 K. At 373 K, the leakage rate increases by about 8%.

The change mentioned above is due to the fact that the viscosity of the lubricant typically decreases as temperature rises, which could contribute to weakened cavitation and viscous shear. Meanwhile, compared with a relatively low speed, viscous shear is stronger, and the influence of changes in thermal-induced cavitation intensity on the opening force seems to decrease at higher speeds. Moreover, thermal cavitation strengthens the pumping flow in the spiral grooves to some extent, which results in increased leakage.

#### 4.1.2. Rotation Speed

**Figure 10:**
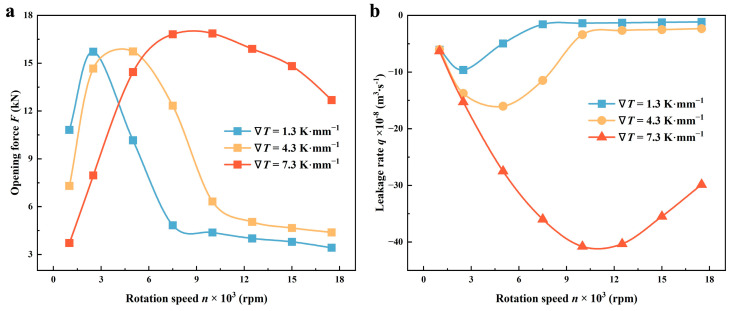
Variations of (**a**) opening force and (**b**) leakage rate as a function of rotation speed *n* under various thermal conditions (*Z* = 12, *h*_g_ = 6 μm, *β* = 18°, *r*_g_ = 32.5 mm, *h*_0_ = 5 μm, *p*_o_ = 0.3 MPa, *T*_i_ = 300 K).

Reiterating the point, the speed at which the seals rotate is a critical factor in determining the extent to which the thermal cavitation effect impacts the seal. Figure 10 further explores the correlation between the performance of seals and rotation speed *n* in various thermal scenarios.

Referring to the work by Adjemout et al. [37], there is a critical speed for the first increasing and then decreasing mass flow rate in a mechanical face seal. The same changing tendency in *F* and *q* could be observed at the three temperature gradients: there is a critical speed for performance change, which shows an increasing trend before the critical speed and a decreasing trend after the critical speed. This is due to the change in both the pumping action of the grooves and the cavitation intensity of the liquid film with the increasing rotation speed.

Moreover, the critical speed value tends to rise as the temperature of the lubricant increases. When the temperature gradient increases from 1.3 to 7.3 K·mm^−1^, the critical speed at which the opening force and leakage rate decrease increases from 2500 rpm to about 10,000 rpm, which means that at high speeds, the lubrication performance of the seal is good, and the sealing characteristics are relatively likely to decline to a certain extent. This is mainly because at 10,000 rpm, the opening force with a temperature gradient of 7.3 K·mm^−1^ is about 3.85 times that of the opening force with a temperature gradient of 1.3 K·mm^−1^, but the leakage rate is about 29 times. This change can be attributed to the occurrence of thermal cavitation. At higher temperatures, the cavitation area becomes more restricted, so an increased velocity has the potential to induce complete cavitation in the grooved region. In the meantime, the decrease in lubricant viscosity results in a reduction of shear impact, which ultimately leads to a decline in both opening force and sealing leakage. When the speed reaches a certain degree, the thermal cavitation effect gradually weakens, and the changes of both gradually tend to be stable.

#### 4.1.3. Seal Pressure

**Figure 11:**
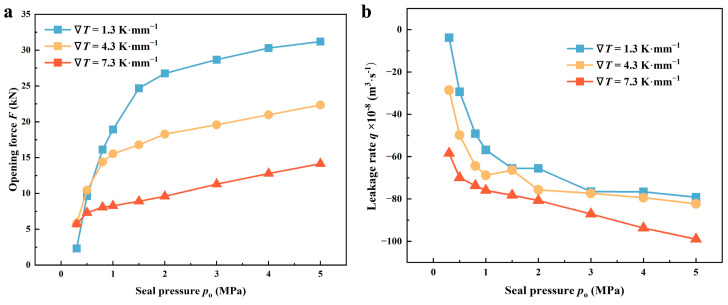
Variations of (**a**) opening force and (**b**) leakage rate as a function of seal pressure *p*_o_ under various thermal conditions (*Z* = 12, *h*_g_ = 6 μm, *β* = 18°, *r*_g_ = 32.5 mm, *h*_0_ = 5 μm, *n* = 10,000 rpm, *T*_i_ = 300 K).

Figure 11 illustrates how the seal performance is affected by the seal pressure *p*_o_ in the thermal cases of 1.3, 4.3, and 7.3 K·mm^−1^. The increased *p*_o_ generally enhances the static opening force and, to some extent, can inhibit liquid film cavitation. As shown in Figure 11a, *F* rises as the seal pressure increases. Nevertheless, the rate of increase in opening force differs across various thermal scenarios. When *p*_o_ increases from 0.3 to 5.0 MPa, the opening force in the cases of 1.3 K·mm^−1^ increases about 13.5 times, and that in the cases of 7.3 K·mm^−1^ increases only by a factor of about 2.5. It can be concluded that thermal cavitation indeed weakens the hydrodynamic performance of the seals. Clearly, for example, when ∇*T* enhances from 1.3 to 7.3 K·mm^−1^, *F* decreases by approximately 56.3% at a pressure of 1.0 MPa. Additionally, as depicted in Figure 11b, the variation in leakage rate *q* closely resembles that of *F*. It is the coupled effect of seal pressure and thermal cavitation that contributes to the decrease of cavitation regions, and then the enhancement of pumping flow. Similarly, when ∇*T* enhances from 1.3 to 7.3 K·mm^−1^, *q* increases by approximately 33.6% at a pressure of 1.0 MPa.

#### 4.1.4. Film Thickness

**Figure 12:**
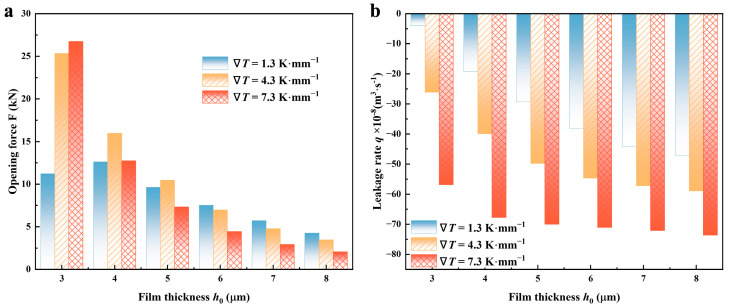
Variations of (**a**) opening force and (**b**) leakage rate as a function of film thickness *h*_0_ under various thermal conditions (*Z* = 12, *h*_g_ = 6 μm, *β* = 18°, *r*_g_ = 32.5 mm, *p*_o_ = 0.3 MPa, *n* = 10,000 rpm, *T*_i_ = 300 K).

Figure 12 illustrates the correlation between sealing performance and film thicknesses at various temperature gradients.

Because the shear effect is impaired at larger film thicknesses, and the thermal cavitation effect will further play negative roles in shear flow, the hydrodynamic effect caused by the accumulation of liquid is correspondingly weakened in the root of the spiral grooves. Therefore, the opening forces under the larger film thicknesses and higher lubricant temperatures usually are smaller, as shown in Figure 12a. For example, when *h*_0_ is 6 mm, the value of F decreases by more than 40% as the temperature gradient of the lubricant enhances to 7.3 from 1.3 K·mm^−1^. For the leakage rate *q*, cavitation in the atmosphere of high temperatures is impeded, which conduces to pump up more lubricant into the micro-gap through grooves. Referred to in Figure 12b, as the gradient of temperature rises to 7.3 from 1.3 K·mm^−1^, the leakage rate increases by over 13 times at *h*_0_ of 3 μm.

### 4.2. Influence of Geometric Configuration

#### 4.2.1. Groove Depth

**Figure 13:**
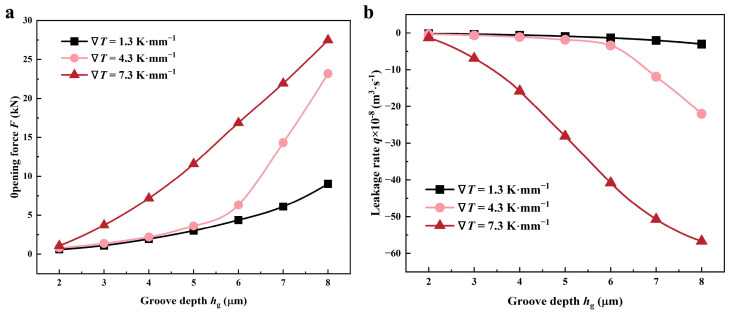
Variations of (**a**) opening force and (**b**) leakage rate as a function of groove depth under various thermal conditions (*Z* = 12, *β* = 18°, *r*_g_ = 32.5 mm, *h*_0_ = 5 μm, *p*_o_ = 0.3 MPa, *n* = 10,000 rpm, *T*_i_ = 300 K).

Figure 13 illustrates the sealing performance variation curve with respect to groove depth *h*_g_ under different thermal conditions. As can be seen from the figures, the depth of the grooves is directly proportional to the increase in both *q* and *F*. As indicated by the experimental work by Brunetiere and Rouillon [48], the temperature rise of the seal face decreased with the increase of groove depth and lubricant temperature. So, when *h*_g_ increases from 2 μm to 8 μm, the ability of the grooves to pump the lubricating medium is enhanced under the coupling effect of grooves configuration and thermal cavitation, which finally improves the hydrodynamic performance. Furthermore, what is noteworthy is that when *h*_g_ is less than 6 μm, the gap in sealing performance is small at ∇*T* = 1.3 and 4.3 K·mm^−1^. After *h*_g_ could be set over that, there is a rapid increase in *F* and *q* for the case of 4.3 K·mm^−1^. At the temperature gradient of 7.3 K·mm^−1^, it is higher than the others in *F* and *q*. Hence, although the thermal cavitation effect of the liquid film at higher temperatures does decrease the hindrance caused by cavitation on pumping efficiency to some degree and improves hydrodynamic performance, it also results in a proportional rise in leakage of the sealed medium.

#### 4.2.2. Groove Number

**Figure 14:**
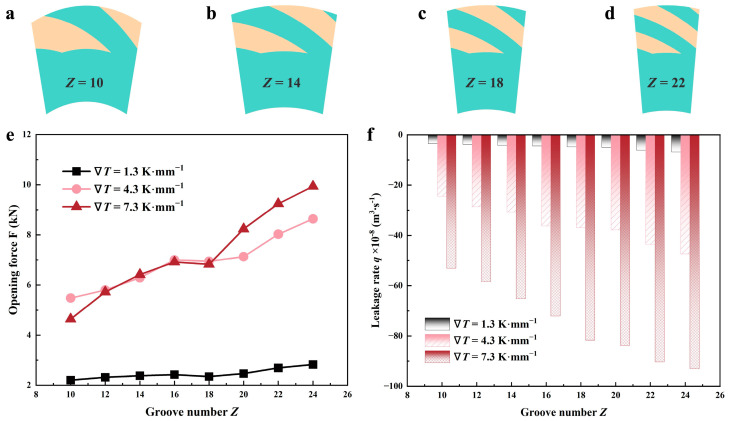
Variations of sealing performance as a function of groove number in different thermal cases (*h*_g_ = 6 μm, *β* = 18°, *r*_g_ = 32.5 mm, *h*_0_ = 5 μm, *p*_o_ = 0.3 MPa, *n* = 10,000 rpm, *T*_i_ = 300 K). (**a**–**d**) show the computational domain at different groove numbers; (**e**,**f**) demonstrate the variability in opening force and leakage rate, correspondingly.

The periodically distributed spiral grooves function as a means of pumping lubricating medium. The correlation between the sealing performance and groove number *Z* in three different thermal cases is depicted in Figure 14. The figure illustrates a clear correlation between the groove number and the rise in both opening and sealing performance. But under the same number of grooves, the temperature change of the lubricating medium is also quite obvious to the sealing performance. With groove number 12, the opening force *F* increases by over 146% when the temperature gradient goes up 6 K·mm^−1^ from 1.3 K·mm^−1^, while there is a gap of only about 1.3% with the temperature increasing by 3 K·mm^−1^ from 4.3 K·mm^−1^. Similarly, the leakage of the lubricant presents a large gap for three thermal cavitation phenomena.

#### 4.2.3. Spiral Angle

**Figure 15:**
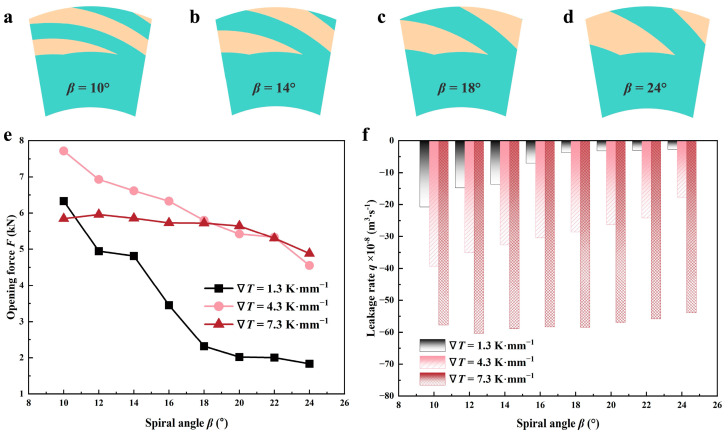
Variations of sealing performance as a function of spiral angle in different thermal cases (*Z* = 12, *h*_g_ = 6 μm, *r*_g_ = 32.5 mm, *h*_0_ = 5 μm, *p*_o_ = 0.3 MPa, *n* = 10,000 rpm, *T*_i_ = 300 K). (**a**–**d**) show the computational domain at different spiral angles; (**e**,**f**) demonstrate the variability in opening force and leakage rate, correspondingly.

Figure 15 demonstrates how the spiral angle *β* affects opening performance and liquid leakage in LFS under lubricant temperature gradients of 1.3, 4.3, and 7.3 K·mm^−1^. The spiral angle increase leads to a decrease in *F* and *q*. Different from the above descriptions, the variation of the opening force in 7.3 K·mm^−1^ tends to be small. When *β* increases from 10 to 24, *F* decreases by about 16.5% in 7.3 K·mm^−1^, but that decreases by approximately 71% in 1.3 K·mm^−1^; analogously, *q* in 1.3 and 7.3 K·mm^−1^ present a decrease of about 86.8% and 7%, respectively.

This is mainly because the increase of *β* under the action of viscous shear will reduce the pumping process, and cavitation is more likely to occur, which will affect the hydrodynamic performance and pumping effect. However, for the thermal cavitation phenomenon at different temperatures, the liquid film viscosity is higher, the cavitation area is relatively large under the condition of small temperature, and the sealing performance is highly responsive to variations in the spiral angle, resulting in a greater reduction in *F* and *q.*

#### 4.2.4. Grooved Radius

**Figure 16:**
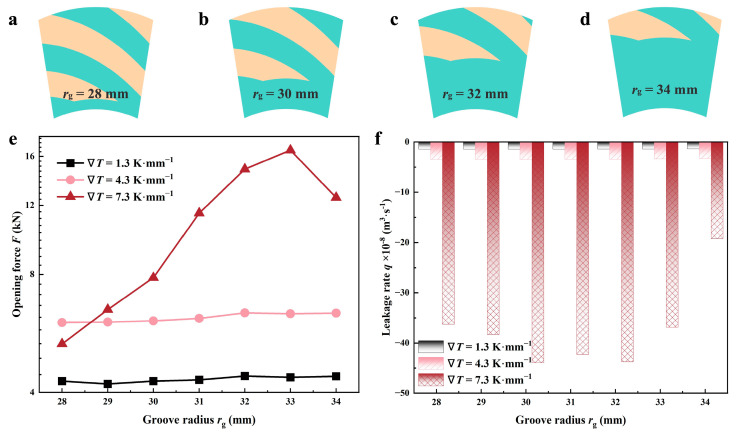
Variations of sealing performance as a function of groove radius in different thermal cases (*Z* = 12, *h*_g_ = 6 μm, *β* = 18°, *h*_0_ = 5 μm, *p*_o_ = 0.3 MPa, *n* = 10,000 rpm, *T*_i_ = 300 K). (**a**–**d**) show the computational domain different groove radii; (**e**,**f**) demonstrate the variability in opening force and leakage rate, correspondingly.

Figure 16 depicts the correlation between groove radius *r*_g_ and sealing performance under various thermal conditions. The opening force in ∇*T* of 7.3 K·mm^−1^ exhibits a distinct variation compared to the values of 1.3 and 4.3 K·mm^−1^, as illustrated in Figure 16e. When the groove radius increases from 28 mm to 34 mm, *F* increases firstly and then gradually declines, which means there is a groove radius to obtain maximum opening force. However, the range of change under a low-temperature gradient is relatively small. For three thermal cases, this value is in the range of 32~33 mm. In Figure 16f, *q* remains almost constant for two cases of relatively low temperature and presents a similar trend with *F* in the case of 7.3 K·mm^−1^.

As can be seen in Figure 16a–d, the different groove radius represents the grooved position of the spiral groove. When the value is smaller, the spiral groove’s root is closer to the inner radius, then the sealing dam area that plays the role of blocking flow and boosting pressure is relatively small. With the increase of *r*_g_, the hydrodynamic effect is strengthened. But, with the increase of temperature gradient, heat leads to the reduction of the cavitation region in the grooves. Under the conditions of 1.3 and 4.3 K·mm^−1^, the cavitation region is still large, and the pumping function of the grooves is still limited. Therefore, the sealing performance changes relatively little with the increase in the groove diameter. However, when the temperature gradient increases to 7.3 K·mm^−1^, the cavitation zone is reduced due to thermal cavitation, so the sealing performance is more significantly affected by the groove radius.

## 5. Concluding Remarks

With the purpose of investigating the impact of thermal cavitation on liquid lubrication performance, a numerical model for evaluating the hydrodynamic characteristics of LFS has been presented when subjected to high speeds and temperature gradients. The main conclusions are shown as follows:

(1) Physical field distribution and cavitation are significantly affected by the thermal cavitation effect. Lubricant temperature gradients can reduce cavitation generation and enhance surface groove pumping. Thermal cavitation reduces the difference in temperature on both sides of the seal face at high speeds. For example, at a speed of 15,000 rpm, the temperature difference decreases from 19.43 K in isothermal conditions to 6.05 K in thermal cases (∇*T* = 3.3 K·mm^−1^), representing a decline of about 68.9%. However, high viscous shear and high-temperature gradients result in elevated surface temperature discrepancies for both sealing rings and liquid film.
(2) The hydrodynamic performance becomes complex at different speeds and temperature gradients due to the thermal cavitation effect. When the lubricant temperature rises from 313 K to 373 K, the opening force decreases by approximately 35.4% at 5000 rpm but increases nearly threefold at 15,000 rpm. In different thermal scenarios, the opening force and leakage will reduce after reaching the critical speed, a higher temperature gradient corresponds to a higher critical speed. Moreover, contrary to the leakage rate under high-temperature gradients, the opening forces tend to be lower with the increasing seal pressure and film thickness.
(3) The hydrodynamic opening performance and leakage exhibit varying trends in different thermal scenarios across a range of geometric configurations. With the increase of the groove depth and groove number, the opening force and leakage rate of the seal show an increasing trend, while the influence of the spiral angle shows an opposite trend. The existence of an optimal groove radius makes the seal obtain a good hydrodynamic effect. Under the corresponding structural parameters, the increase in temperature gradient will increase the opening force and leakage rate. The implication here is that when designing geometric grooves, the thermal cavitation effect should be considered to achieve improved hydrodynamic performance.

Above all, the thermal cavitation could impact the occurrence of the cavitation and then the performance of the seals. In the future theoretical analysis and engineering applications, this study may provide insightful information for practical sealing design.

## Figures and Tables

**Table 1:** Geometric configuration parameters for LFS.

Geometric Configuration	Notation	Data
Outer radius	*r*_o_ (mm)	37
Inner radius	*r*_i_ (mm)	27
Spiral groove	*β* (°)	10~24
Number of grooves	*Z*	10~24
Grooved radius	*r*_g_ (mm)	28~34
Depth of grooves	*h*_g_ (μm)	2~9

**Table 2:** Working Parameters for LFS.

Working Parameters	Notation	Data
Medium temperature	*T*_0_ (K)	273~373
Seal pressure	*p*_o_ (MPa)	0.3~5
Inner pressure	*p*_i_ (MPa)	0.1
Designed film thickness	*h*_0_ (μm)	3~8
Rotation speed	*n* (rpm)	1000~20,000

**Table 3:** Properties of lubricating medium for LFS.

Properties	Notation	Data
Liquid density	*ρ*_0_ (kg·m^−3^)	800
Liquid viscosity	*η*_0_ (mPa·s)	17.9
Cavitation pressure	*p*_c_ (kPa)	30
Heat conduction	*K*_c_liq_ (W·m^−2^·K^−1^)	0.12
Specific heat	*c*_p_ (J·kg^−1^·K^−1^)	1870

**Table 4:** Material properties for the sealing ring of LFS.

Material Properties	Notation	Stainless Steel	Graphite Ring
Density	*ρ*_s_ (kg·m^−3^)	7800	1800
Elastic coefficient	*E* (MPa)	204,000	25,000
Poisson ratio	*υ*	0.3	0.2
Heat conduction	*k*_c_ (W·m^−2^·K^−1^)	16.5	15
Specific heat	*c*_s_ (J·kg^−1^·K^−1^)	460	710
Linear thermal expansion	*l* (K^−1^)	15.9 × 10^−6^	4 × 10^−6^

## Data Availability

Data are contained within the article.

## Nomenclature

LFS	Liquid face seal	*q*_r_	Radial flow rates
*r*_i_	Inner radius	*η*_0_	Lubricant viscosity in initial state
*T*_i_	Inside medium temperature	*τ*	Viscosity–temperature coefficient
*p*_i_	Inner pressure	*k*_c_liq_	Fluid’s heat conduction
*h*_g_	Groove depth	*c*_p_	Specific heat
*T*_0_	Outside medium temperature	*ρ*_s_	Sealing ring’s density
*p*_o_	Outer pressure	*k*_c_	Sealing ring’s heat conduction
*r*_o_	Outer radius	*c*_s_	Sealing ring’s specific heat
*n*	Rotation speed	*E*	Elastic coefficient
*h*	Local film thickness	*υ*	Poisson ratio
*C*_s_	Stator thickness	*l*	Linear thermal expansion
*C*_r_	Rotor thickness	*q*	Leakage rate
*β*	Spiral groove	*F*	Opening force
*Z*	Groove number	*P*	Dimensionless pressure
*r*_g_	Groove radius	*p*_a_	Ambient temperature
*h*	Liquid viscosity	*z*	Axial direction
*r*	Liquid density	*i*	Grid note in *q* direction
*ω*	Angular velociry	*j*	Grid note in *r* direction
*p*	Local pressure	*k*	Grid note in *z* direction
*r*	Radial direction	*T*_av_	Average temperature
*θ*	Circumsferential direction	*ε*	Error limit
*S*	Cavitation intensity	*Y*	Iteration parameters
*p*_c_	Cavitation pressure	*m*	Number of iterations
*ρ*_0_	Fluid critical density	∇*T*	Temperature gradient
*p*_min_	Minimum pressure	*P*_max_	Maximum temperature
*T*_s_	Seal ring temperature	∆*T*	Temperature difference
*T*	Local temperature	Subscripts	
*k*_ls_	heat transfer convection	1	Rotating ring
*c*_v_	lubricant’s specific heat	2	Stationary ring
*q*_θ_	Circumferential flow rates		
